# Supplementary material for: Farmers’ Adaptive Behaviors to Heavy Metal-Polluted Cultivated Land in Mining Areas: The Influence of Farmers’ Characteristics and the Mediating Role of Perceptions
Source: Int J Environ Res Public Health. 2022 May 31;19(11):6718. doi: 10.3390/ijerph19116718 (PMC9180364; doi:10.3390/ijerph19116718)
Supplement: Supplementary file 1 [file ijerph-19-06718-s001.zip › ijerph-1739632-supplementary.pdf]

# Supplementary Materials:

**Table S1.** Description and measurement of farmers' perceptions

| Farmers' perceptions | | **Variable description** | **Scale** | **Mean** | **Std. Dev.** |
|---|---|---|---|---|---|
| Loss perception | Farming impact (LossP-F) | Do you think HMP affects the yield and quality of agricultural products? | Each question is scored with an integer value between 1 and 5, where 1 represents the lowest order and 5 represents the highest order. | 3.68 | 1.65 |
| | Health impact (LossP-H) | Do you think HMP affects people's health? | | 3.97 | 1.28 |
| Adaptation perception | Adaptive efficacy (AdaptP-E) | Do you think your behaviors can effectively mitigate HMP? | | 2.52 | 1.15 |
| | Self-efficacy (AdaptP-S) | Do you have the ability to control HMP in cultivated land? | | 1.58 | 0.73 |
| | Adaptive cost (AdaptP-C) | Are you willing to spend time and money on the treatment of HMP? | | 2.89 | 1.73 |
